# Cytotoxic, tubulin-interfering and proapoptotic activities of 4′-methylthio-*trans*-stilbene derivatives, analogues of *trans*-resveratrol

**DOI:** 10.1007/s10616-018-0227-3

**Published:** 2018-05-28

**Authors:** Renata Mikstacka, Małgorzata Zielińska-Przyjemska, Zbigniew Dutkiewicz, Michał Cichocki, Tomasz Stefański, Mariusz Kaczmarek, Wanda Baer-Dubowska

**Affiliations:** 1 Department of Inorganic and Analytical Chemistry, Nicolaus Copernicus University, Ludwik Rydygier Collegium Medicum, Dr A. Jurasza 2, 85-089 Bydgoszcz, Poland; 2 Department of Chemical Technology of Drugs, Poznań University of Medical Sciences, Grunwaldzka 6, 60-780 Poznań, Poland; 3 Department of Pharmaceutical Biochemistry, Poznań University of Medical Sciences, Święcickiego 4, 60-781 Poznań, Poland; 4 Department of Clinical Immunology, Poznań University of Medical Sciences, Rokietnicka 5d, 60-806 Poznań, Poland

**Keywords:** Apoptosis, Cytotoxicity, Resveratrol, Stilbenes, Tubulin polymerization

## Abstract

The aim of this study was to evaluate the cytotoxicity of a series of seven 4′-methylthio-*trans*-stilbene derivatives against cancer cells: MCF7 and A431 in comparison with non-tumorigenic MCF12A and HaCaT cells. The mechanism of anti-proliferative activity of the most cytotoxic *trans*-resveratrol analogs: 3,4,5-trimethoxy-4′-methylthio-*trans*-stilbene (3,4,5-MTS) and 2,4,5-trimethoxy-4′-methylthio-*trans*-stilbene (2,4,5-MTS) was analyzed and compared with the effect of *trans*-resveratrol. All the compounds that were studied exerted a stronger cytotoxic effect than *trans*-resveratrol did. MCF7 cells were the most sensitive to the cytotoxic effect of *trans*-resveratrol analogs with IC_50_ in the range of 2.1–6.0 µM. Comparing the cytotoxicity of 3,4,5-MTS and 2,4,5-MTS, a significantly higher cytotoxic activity of these compounds against MCF7 versus MCF12A was observed, whereas no significant difference was observed in cytotoxicity against A431 and HaCaT. In the series of 4′-methylthio-*trans*-stilbenes, 3,4,5-MTS and 2,4,5-MTS were the most promising compounds for further mechanistic studies. The proapoptotic activity of 3,4,5-MTS and 2,4,5-MTS, estimated with the use of annexin-V/propidium iodide assay, was comparable to that of *trans*-resveratrol. An analysis of cell cycle distribution showed a significant increase in the percentage of apoptotic cells and G2/M phase arrest in MCF7 and A431 as a result of treatment with 3,4,5-MTS, whereas *trans*-resveratrol tended to increase the percentage of cells in S phase, particularly in epithelial breast cells MCF12A and MCF7. Both *trans*-stilbene derivatives enhanced potently tubulin polymerization in a dose-dependent manner with sulfur atom participating in the interactions with critical residues of the paclitaxel binding site of β-tubulin.

## Introduction

Resveratrol (3,4′,5-trihydroxy-*trans*-stilbene), a naturally occurring phytoalexin, is the most extensively studied stilbene derivative. This compound has been shown to exert several beneficial effects, including cancer chemopreventive activity (Athar et al. 2009; Vang et al. 2011; Varoni et al. 2016; Jiang et al. 2017). Molecular mechanisms of anticancer properties of resveratrol concern three stages of carcinogenesis: initiation, promotion and progression. Resveratrol exerts the suppressive activity on extracellular growth factors, receptor tyrosine kinases and cytoplasmic tyrosine signalling pathways. It influences redox status of cells and demonstrates anti-inflammatory and anti-estrogenic activities; and the inhibitory effect on activation of procarcinogens catalysed by cytochrome P450 family 1 enzymes. Resveratrol inhibits cell proliferation by modulating the expression of cyclins and cyclin-dependent kinases (CDKs) and CDK inhibitors (Varoni et al. 2016). The induction of apoptosis is considered to be one of the most essential among possible mechanisms of its chemopreventive and potentially chemotherapeutic activity. Resveratrol has been shown to stimulate an apoptotic pathway in various types of cells (Wang et al. 2010; Taguchi et al. 2016). It was also shown that resveratrol has the potential to increase sensitivity to apoptotic stimuli by suppressing the expression of anti-apoptotic molecules in ovarian cancer cells (Taguchi et al. 2016). Apoptotic pathways are usually activated when the cell cycle cannot be restored (Moeller and Sheaff 2006). Moreover, cancerous cells exhibit high growth rates because of cell cycle and apoptosis deregulation. Thus, induction of cell cycle arrest is considered a rational strategy to turn tumor cells into apoptotic death (Schwarz and Shah 2005).

Tubulin monomers polymerize to form microtubules that are major components of the cytoskeleton. At the beginning of mitosis, the interphase microtubular network forms a mitotic spindle that segregates the replicated chromosomes into daughter cells. Certain anti-cancer drugs cause suppression of microtubule dynamics either by inhibiting polymerization (e.g. colchicine, vinblastine, vincristine, vinorelbine) or by enhancing it (e.g. paclitaxel, docetaxel), driving the cells into apoptosis (Mollinedo and Gajate 2003; Singh et al. 2008; Nagle et al. 2012). Stilbenes, which are microtubule interfering agents (MIA), draw attention in the context of the search for potent cancer remedies. Many natural MIAs have been identified recently, and still new classes of potential MIA are synthesized; among them, potent antitubulin agents are selected (Kamal et al. 2015, 2016; Madadi et al. 2015).

Resveratrol has been shown to induce apoptosis and/or cell cycle arrest, among others in breast cancer MCF7 cells (Su et al. 2013) and human epidermoid carcinoma A431 cells (Ahmad et al. 2001). An important issue related to the future application of resveratrol in disease management is its low bioavailability due to its rapid metabolism, mainly sulphation and glucuronidation in mammals (Walle et al. 2004). The problem that remains still unresolved is the dose of resveratrol most suitable for effective cancer preventive intervention. Mechanistic studies in cells in vitro have almost invariably used concentrations of resveratrol in the 10^−5^–10^−4^ M range, which is much higher than those which can be achieved in humans. Thus, new experimental paradigms need to be used to obtain information on pharmacological changes elicited by resveratrol when present at very low concentrations or when administered at dietary-relevant doses (Scott et al. 2012). Recent multicenter studies indicated that the low doses of chemicals may act efficiently, and this phenomenon is explained by cumulative effect of chemicals acting on different pathways and mechanisms (Goodson et al. 2015).

A natural analog of resveratrol, 3,4′,5-trimethoxy-*trans*-stilbene which possesses substituents that could not be conjugated with sulphuric or glucuronic acid exerts a stronger proapoptotic effect in cancer cells (Wang et al. 2010). A strategy focused on discovering and defining novel analogs of resveratrol has been introduced. The analogs should have the same structural backbone of resveratrol, with chemical modifications resulting in superior efficacy (Szekeres et al. 2010; Chimento et al. 2016). Besides expected higher bioavailability, such new derivatives may be potentially used in combination with the parent compound or the other analogs. Such an approach when anticancer drug combination are simultaneously targeting different pathways has been suggested as more effective, not only in therapy but also in chemoprevention (Block et al. 2015).

Our earlier studies showed that the introduction of the methylthio-group into the stilbene core may influence the efficacy and selectivity of the inhibitory potency of these compounds toward the P450 isozymes (Mikstacka et al. 2012; Szaefer et al. 2014), as well as enhance the expression of phase II enzymes controlled by Nrf2 transcription factor in vivo and in vitro (Krajka-Kuźniak et al. 2014). These observations suggested that methylthio derivatives of stilbenes may protect the cells, better than resveratrol does, against reactive electrophilic metabolites of some carcinogens. On the other hand, other authors showed that substitution of the 4′ oxygen with a less electronegative sulphur atom also reduced toxicity toward the HEK 293 cells, but enhanced the compound’s ability to activate human SIRT1 (Yang et al. 2007). Activation of SIRT1 may contribute to reduced apoptosis and lead to cancer phenotype. In this regard, it was shown that SIRT1 activation is involved in benzo[a]pyrene induced lung tumorigenesis (Lu et al. 2015).

In order to better characterize a potential anti-carcinogenic activity of stilbene methylthio-derivatives, in this study the effect of the two most potent analogues of resveratrol: 3,4,5-trimethoxy-4′-methylthio-*trans*-stilbene (3,4,5-MTS) and 2,4,5-trimethoxy-4′-methylthio-*trans*-stilbene (2,4,5-MTS) on cell cycle viability, apoptosis and cell cycle distribution in breast and skin cancer cells, and their non-tumorigenic counterparts was evaluated. Besides, the effect of 3,4,5-MTS and 2,4,5-MTS on tubulin polymerization in vitro was investigated. Molecular docking was employed to characterize the specificity of interactions of 3,4,5-MTS and 2,4,5-MTS with β-tubulin.

## Materials and methods

### Chemicals

Resveratrol (purity 99%), antibiotic solution (10,000 units’ penicillin, 10 mg streptomycin and 25 μg amphotericin B per mL), camptothecin, dimethyl sulfoxide (DMSO), fetal bovine serum (FBS), Dulbecco’s Modified Eagle’s Medium (DMEM), Ham’s F12 mixture, propidium iodide (PI), ribonuclease A (RNase A) and 3-(4,5-dimethylthiazol-2-yl)-2,5-diphenyltetrazolium bromide (MTT) and all other compounds were provided by Sigma-Aldrich Co. (St Louis, MO, USA).

Polymethoxy 4′-methylthio-*trans*-stilbenes were synthesized as described previously (Mikstacka et al. 2012). Their structures are shown in Table 1.

### Cell culture and treatment

Spontaneously immortalized human keratinocyte HaCaT cells were purchased from Cell Lines Service (CLS, Eppelheim, Germany). Human epidermoid carcinoma A431 cells were obtained from Deutsche Sammlung von Mikroorganismen und Zellkulturen (DSMZ, Braunschweig, Germany). MCF7 (ECACC 86012803) and MCF12A (ECACC10782) cells were purchased from the European Collection of Cell Cultures (Salisbury, Wiltshire, UK). MCF12A cells were cultured in 95% 1:1 mixture of DMEM and Ham’s F12 medium, 20 ng/mL human epidermal growth factor, 500 ng/mL hydrocortisone and 5% horse serum. The other cell lines were cultured in Dulbecco’s modified Eagle’s medium (DMEM) supplemented with 10% fetal bovine serum and 1% (v/v) antibiotics solution. Cells were incubated at 37 °C in an atmosphere consisting of 95% air and 5% CO_2_ in a humidified incubator until they reached 70% confluency. 1 × 10^6^ cells were seeded in 40 mm ø culture dishes. After 24 h of preincubation in DMEM containing 5% of FBS, the cells were treated with resveratrol or its analogs, and the incubation was continued for a subsequent 24 h to assess apoptosis or cell cycle distribution. Then, the cells were harvested. Control cells were treated with DMSO, at a concentration of less than 0.1%.

### Cell viability assay

The effect of resveratrol and 4′-methylthiostilbenes on cell viability was assessed with MTT assay according to the standard protocol described earlier (Zielińska-Przyjemska et al. 2015). Briefly, the cells were seeded in 96-well plates at a density of 1 × 10^4^ cells/well in 100 μL of growth medium. They were allowed to attach overnight and either resveratrol or the appropriate analog was then added to the culture medium at various concentrations (0–200 μM) for 48 h at 37 °C. The cells were subsequently incubated with MTT (0.5 mg/mL) solution for another 4 h. The water insoluble formazan crystals were solubilized in acidic isopropanol before the measurement of absorbance using a microplate reader (TECAN Infinite M200, TK Biotech, Warsaw, Poland) at 540 and 690 nm. All of the experiments were repeated three times, with at least three measurements per assay.

### Apoptosis/necrosis determination: Annexin-V/propidium iodide assay

Apoptosis and necrosis were detected using Annexin-V-FLUOS Staining Kit assay (Roche Diagnostics GmbH, Mannheim, Germany), according to the manufacturer’s instruction. After have been treated with test compounds for 24 h, the cells were transferred (1 × 10^6^ cells in 100 µL of the solution) into 5 mL culture tubes followed by the addition of 2 µL of Annexin-V-Fluos and 2 µL PI. Camptothecin at a final concentration of 50 nM was used as a positive control. Samples were gently mixed and incubated for 15 min at RT (25 °C) in the dark. Fluorescence of cell surface (AV) and DNA-bound PI markers was analyzed with flow cytometry (Becton–Dickinson, San Jose, CA, USA) at 488 nm excitation wavelength, emission 518 and 617 nm for AV and PI, respectively.

### TUNEL assay

TUNEL assay was applied to detect apoptotic cells using In Situ Cell Death Detection Kit (Roche Diagnostics, Indianapolis, IN, USA). Briefly, after incubation with the test compounds for 24 h, the cells were detached with a 0.5% trypsin–EDTA solution and collected. Cell suspensions were fixed with 4% paraformaldehyde and permeabilized with 0.1% Triton X-100 in 0.1% sodium citrate. After the TUNEL reaction mixture was added, cells were incubated for 1 h at 37 °C in a humidified chamber and samples were analyzed by FACSCanto Flow Cytometer (Becton–Dickinson). Camptothecin at a final concentration of 50 nM was used as a positive control.

### Flow cytometry cell cycle analysis

The cells harvested after a 24 h incubation with the test compounds were washed with 1 mL of PBS and fixed with 70% ethanol. The ethanol was added dropwise to the cell pellet while vortexing to ensure fixation of all cells and minimizing clumping. After a 30 min incubation, the cells were washed twice in PBS, and 250 µL of a solution containing 50 µg/mL PI, 100 µg/mL RNase A (Sigma, St. Louis, MO, USA) in PBS was added to the pellet and incubated for 30 min at 37 °C in the dark. The stained cells were analyzed by FACSCanto Flow Cytometer (Becton–Dickinson). Camptothecin at a final concentration of 50 nM was used as a positive control. Data analysis and acquisition were performed using FACS Diva software (Becton–Dickinson).

### Tubulin polymerization assay

Tubulin polymerization was assessed with the use of purified porcine tubulin purchased from Cytoskeleton Inc. (Denver, CO, USA) in accordance with a protocol recommended by the manufacturer. Tubulin was dissolved in a buffer containing: 80 mM PIPES pH 6.9, 2 mM MgCl_2_, 0.5 mM EGTA and 1 mM GTP, at a final concentration of 3 mg/mL and placed in a 96-well plate (0.3 mg per well). The polymerization reaction was started by increasing temperature from 4 to 37 °C upon transfer of the reaction mixture to a pre-warmed plate. The assembly of microtubules was monitored spectrophotometrically by measuring absorbance at 350 nm for 60 min, at a temperature of 37 °C. Paclitaxel was used as stabilizing positive control.

### Molecular docking

3,4,5-MTS, 2,4,5-MTS and paclitaxel were docked to the paclitaxel binding site of tubulin (PDB: 1JFF) (Lowe et al. 2001) by applying BIOVIA Discovery Studio 2016 (Dassault Systèmes BIOVA 2016) CDOCKER procedure (Wu et al. 2003). Docking was performed with the use of CHARMm force field with Momany-Rone charges for both the receptor and ligands. The binding site was defined around the centroid of the cocrystallized ligand (paclitaxel). The radius of the sphere was set to the value of 12 Å. For each ligand, 20 initial conformations were generated randomly using high-temperature grid-based molecular dynamics, and then translated into the binding site. Twenty orientations of each conformation were used for docking and further simulated annealing and minimization. Ten distinct binding complexes obtained by the docking search technique were subjected to energy ranking and protein–ligand interactions analysis.

### Statistical analysis

All results obtained in the experiments with cells in vitro were presented as mean values ± SEM of three independent experiments. Differences were considered significant for *p* values less than 0.05. The cell viability, cell cycle and apoptosis data were evaluated by analysis of variance (ANOVA) followed by Dunnett’s post hoc test using GraphPad Prism Version 4.03 Sofware (San Diego, CA, USA).

## Results

### The effect of resveratrol and 4′-methylthio-stilbenes on cell viability

We determined IC_50_ values for a 48 h treatment of human non-tumorigenic (MCF12A and HaCaT) and cancer derived (MCF-7 and A431) cell lines with resveratrol and its analogs (Table 1). Only 3,4,5-MTS and 2,4,5-MTS exhibited higher cytotoxity against all cell lines tested when compared to the parent compound. Moreover, similarly to resveratrol, 2,4,5-MTS reduced the viability of cancer cells (MCF7, A431) to a greater extent than their non-tumorigenic counterpart cells (MCF12A and HaCaT). The 3,4,5-MTS exhibited the highest cytotoxicity toward MCF7 cells, while IC_50_ of 2,4,5-MTS was lower than that of 3,4,5-MTS in MCF12A cells. The MCF7 cells were the most sensitive to the cytotoxic activity of all studied stilbene derivatives with IC_50_ ranging between 2.1 and 27.3 µM.

Based on the results of viability assay, the concentrations of the tested compounds used in the further studies included values lower and higher than IC_50_.

**Table 1:** The effect of resveratrol and 4′-methylthiostilbenes^a^ on cell viability (IC_50_; µM) 
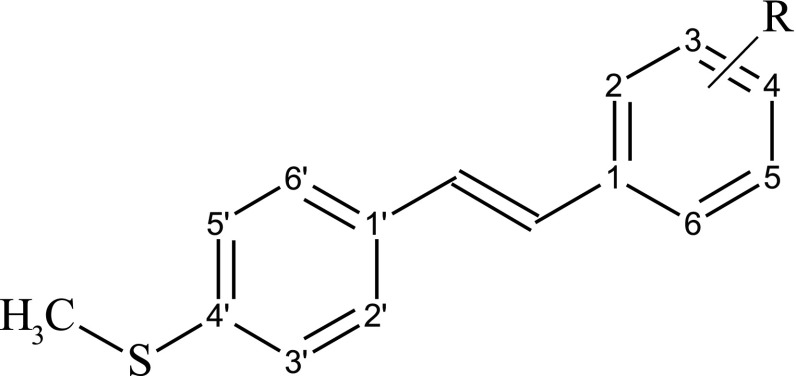

Tested compounds	IC_50_ (µM)			
	MCF12A	MCF7	HaCaT	A431
Resveratrol (*trans*-3,4′,5-trihydroxystilbene)	93.5 ± 12.1	24.6 ± 3.8	85.2 ± 29.2	49.4 ± 3.8
2-Methoxy-4′-methylthio-*trans*-stilbene	n.d.	27.3 ± 12.4	106.4 ± 9.3	241.3 ± 10.0
3-Methoxy-4′-methylthio-*trans*-stilbene	n.d.	6.6 ± 3.7	182.7 ± 38.7	141.3 ± 2.8
2,3-Dimethoxy-4′-methylthio-*trans*-stilbene	n.d.	10.6 ± 6.1	51.8 ± 7.3	157.3 ± 3.0
2,5-Dimethoxy-4′-methylthio-*trans*-stilbene	n.d.	10.2 ± 6.6	65.0 ± 24.5	47.1 ± 9.0
3,5-Dimethoxy-4′-methylthio-*trans*-stilbene	n.d.	4.8 ± 3.4	25.4 ± 13.9	45.4 ± 7.2
3,4,5-Trimethoxy-4′-methylthio-*trans*-stilbene (3,4,5-MTS)	25.7 ± 4.3	2.1 ± 0.4	10.0 ± 4.6	19.3 ± 5.5
2,4,5-Trimethoxy-4′-methylthio-*trans*-stilbene (2,4,5-MTS)	10.0 ± 3.0	5.1 ± 1.4	50.9 ± 13.3	22.6 ± 7.2

*n.d.* not determined

^a^The structure of studied compounds is displayed with general formula, where R represents metoxy substituents linked to phenyl ring of stilbene

### The effect of resveratrol and 4′-methylthio-stilbenes on cell cycle distribution

**Fig. 1:**
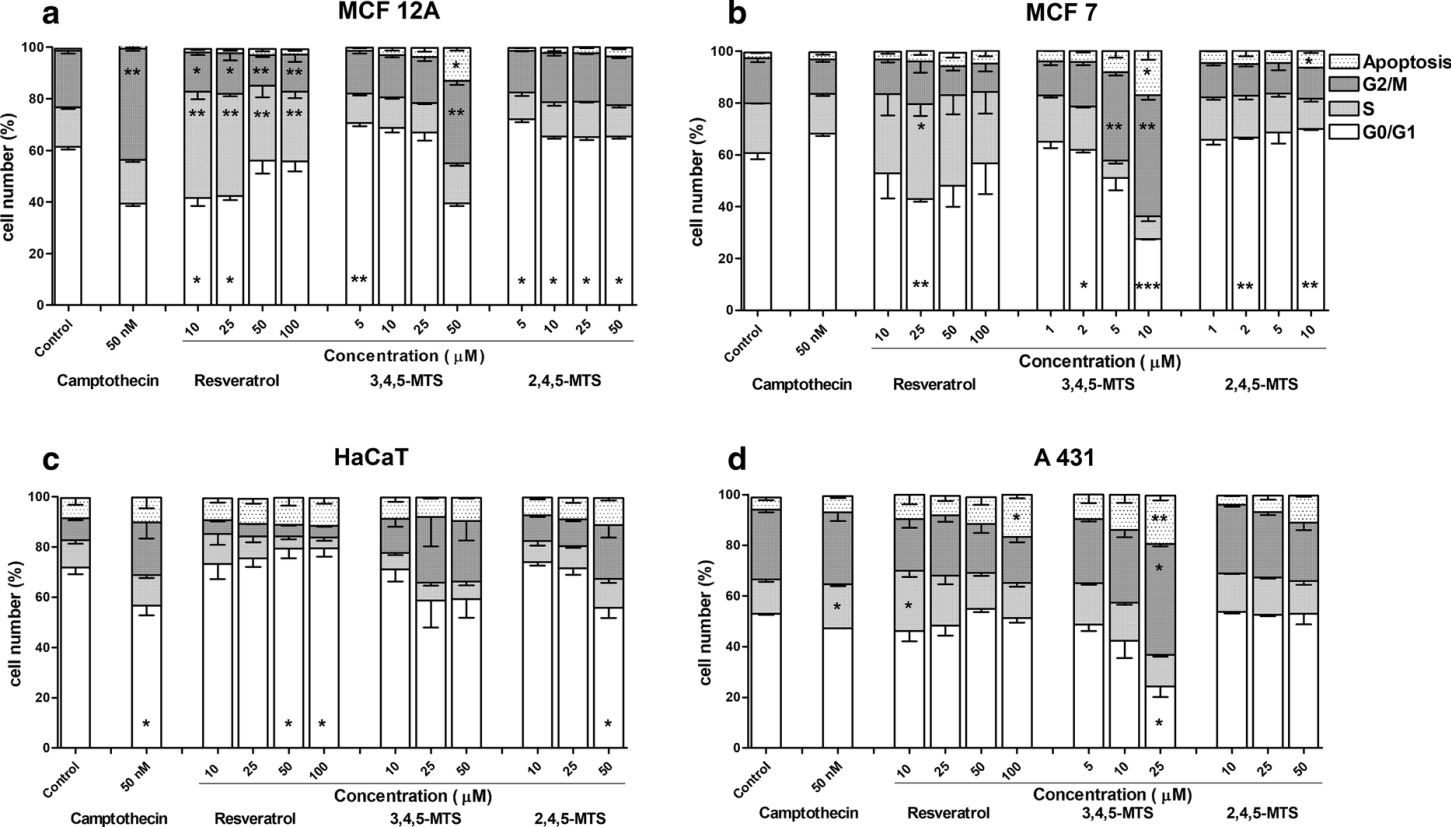
Cell cycle distribution in MCF12A (**a**), MCF7 (**b**), HaCaT (**c**) and A431 (**d**) cell lines after 24 h of incubation with resveratrol, 3,4,5-MTS and 2,4,5-MTS followed by propidium iodide labeling and flow cytometry analysis. Camptothecin at final concentration of 50 nM was used as a positive control. Results of three independent experiments are presented as mean ± SEM. *p < 0.05, **p < 0.01, ***p < 0.001 compared to untreated control using ANOVA followed by Dunnett’s test

Flow cytometric cell cycle analysis was used in order to assess the effect of resveratrol and 4′-methylthio-stilbenes on cell cycle distribution (Fig. 1). The analysis of cell cycle distribution confirmed the proapoptotic effect of resveratrol and methylthio-stilbenes, particularly in MCF7 and A431cells (Fig. 1). The percentage of apoptotic cells in both cell lines increased in a dose dependent manner as a result of treatment with 3,4,5-MTS and 2,4,5-MTS. In normal immortalized cells (MCF12A and HaCaT), this effect did not occur. Moreover, 3,4,5-MTS induced G2/M phase arrest which was statistically significant in MCF12A, MCF7 and A431 cells. This effect was not observed for 2,4,5-MTS, whereas, in MCF12A and MCF7 cells, 2,4,5-MTS- induced G0/G1 phase arrest was shown. Resveratrol at low concentrations tended to increase the percentage of cells in S phase as compared with control, particularly in non-tumorigenic MCF-12 and cancer derived MCF7 cells, while for higher concentrations the percentage of cells in G0/G1 was increasing with a simultaneous decrease of the fraction of cells in S phase.

### The effect of resveratrol and 4′-methylthio-stilbenes on apoptosis induction

Proapoptotic activity of resveratrol and 4′-methylthio-stilbenes was estimated with the use of Annexin-V affinity assay based on double staining. Phosphatidylserine externalization occurs in early apoptotic stages of apoptosis as a result of lost membrane integrity. Detection of phospholipid externalization can be achieved by conjugation with the labeled annexin-V (Annexin-V-Fluos), while, propidium iodide, the DNA binding dye enters only dead cells with a permeable membrane; thus this double staining discriminates viable cells from dead and/or apoptotic cells.

**Fig. 2:**
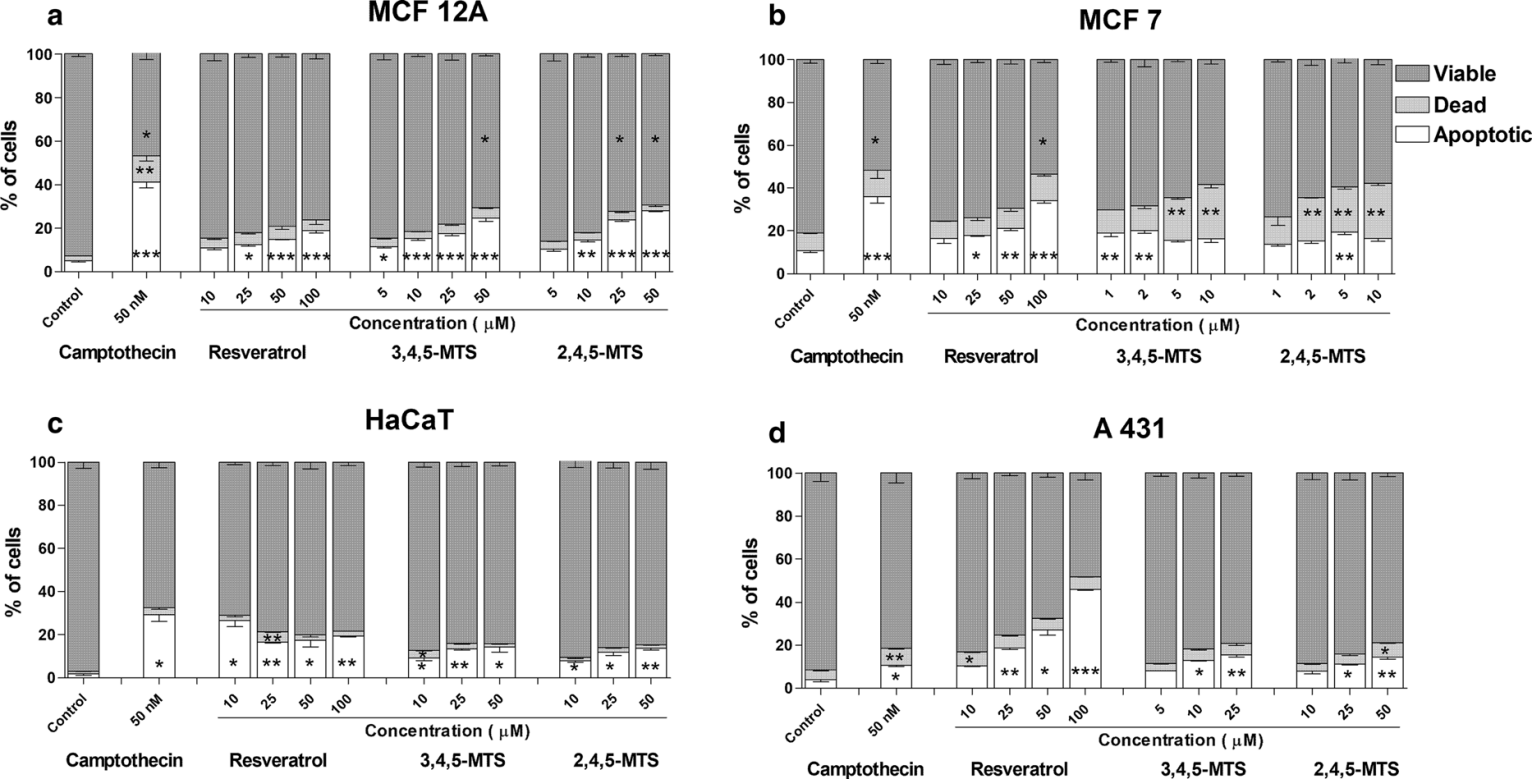
Effect of resveratrol, 3,4,5-MTS and 2,4,5-MTS on phosphatidylserine externalization and propidium iodide staining in MCF12A (**a**), MCF7 (**b**), HaCaT (**c**) and A431 (**d**) cell lines after 24 h of incubation subjected to Annexin-V binding and propidium iodide labeling followed by flow cytometry analysis. The cell populations of AV−/PI−, AV+/PI+ and AV+/PI− representing viable, dead and apoptotic cells, respectively, were estimated. Camptothecin at final concentration of 50 nM was used as a positive control. Results of three independent experiments are presented as mean ± SEM. *p < 0.05, **p < 0.01, ***p < 0.001 compared to untreated control using ANOVA followed by Dunnett’s test

As shown in Fig. 2, apoptosis was evident in all the tested cell lines as a result of treatment with resveratrol or its derivatives. In MCF12A, MCF7 and A431 cell lines, resveratrol was an efficient inducer of apoptosis, and this effect was dose dependent. In HaCaT cells, the effect of resveratrol and its derivatives was significant. However, the amount of apoptotic cells did not change with an increasing concentration of the compounds studied. 3,4,5-MTS and 2,4,5-MTS in the range of concentrations used induced apoptosis in all the cell lines. In MCF7 cells, the proapoptotic effect was assessed for concentrations below 10 µM because of a high cytotoxicity of 3,4,5-MTS and 2,4,5-MTS; 2.1 and 5.1 µM, respectively. In these cells, a significantly higher percentage of dead cells was observed as a result of treatment with 3,4,5-MTS and 2,4,5-MTS.

**Fig. 3:**
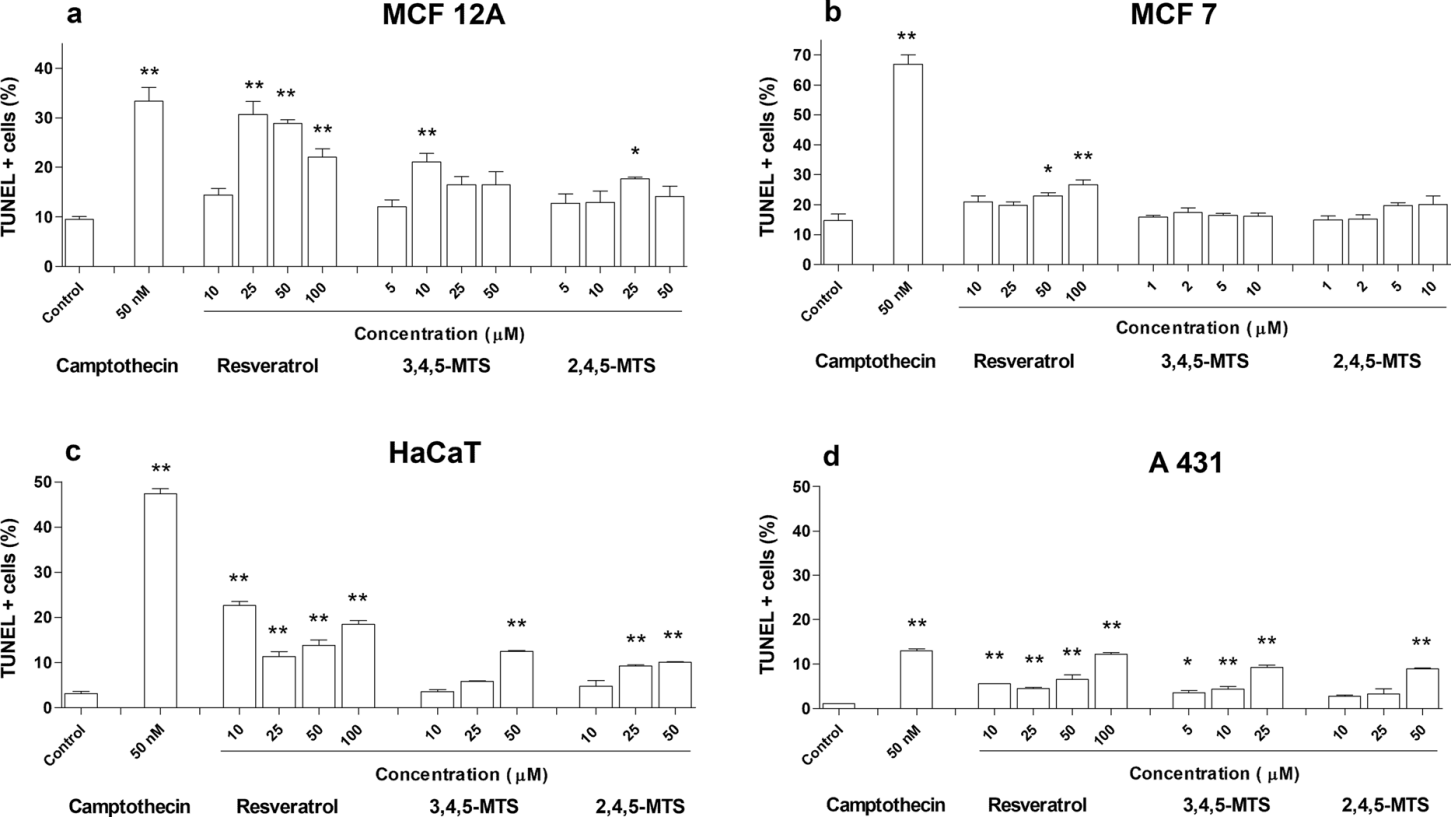
DNA fragmentation in MCF12A (**a**), MCF7 (**b**), HaCaT (**c**) and A431 (**d**) cell lines assessed by TUNEL test using terminal transferase and flow cytometry analysis after 24 h of incubation with resveratrol, 3,4,5-MTS and 2,4,5-MTS. Camptothecin at final concentration of 50 nM was used as a positive control. Results of three independent experiments are presented as mean ± SEM. *p < 0.05, **p < 0.01 compared to untreated control using ANOVA followed by Dunnett’s test

TUNEL assay was employed to estimate the proapoptotic properties of the studied compounds in the late stages of apoptosis. The results of TUNEL assay in the studied range of concentrations did not show a significant dose dependence (Fig. 3). However, an increase in the number of TUNEL positive cells in relation to the concentrations of the compounds studied was partially observed in HaCaT and A431 cells. The effect of resveratrol on the number of TUNEL positive cells in relation to the control value occurred in all the studied cell lines, with the highest percentage of TUNEL positive cells observed in MCF12A cells. The effect of 3,4,5-MTS and 2,4,5-MTS was noticeable in all cell lines, although a statistically significant increase in the number of TUNEL positive cells was observed only in HaCaT and A431 when higher concentrations of compounds were added.

### The effect of resveratrol and 4′-methylthio-stilbenes on tubulin polymerization in vitro

**Fig. 4:**
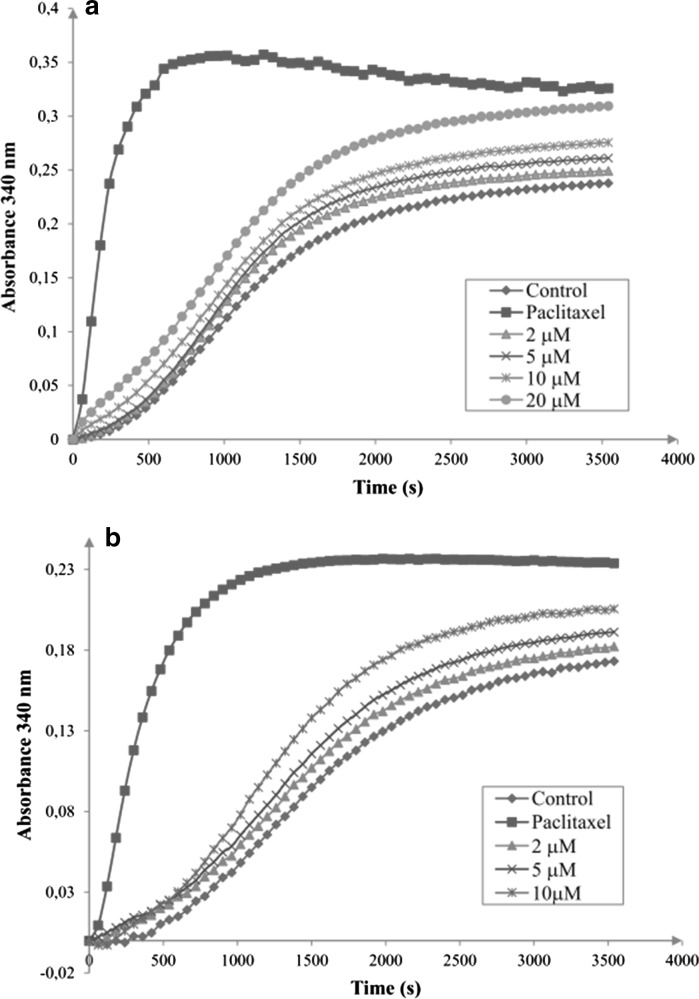
Effect of 3,4,5-MTS (**a**) and 2,4,5-MTS (**b**) on tubulin polymerization. Purified tubulin protein (cytoskeleton) in PIPES buffer containing 1 mM GTP were incubated at 37 °C in the absence (control) and presence of studied compounds. Turbidity was measured at 340 nm in 2 min intervals during 60 min. As a positive standard 10 µM paclitaxel was used. Each point represents an average of two independent samples

The anti-proliferative activity of 3,4,5-MTS and 2,4,5-MTS prompted us to investigate the effect of the studied compounds on microtubule organization. The effect of 3,4,5-MTS and 2,4,5-MTS on tubulin polymerization was determined in vitro and compared with paclitaxel used as a positive control. Both compounds influenced tubulin polymerization in a concentration-dependent manner (Fig. 4a, b). Absorbance measured during the polymerization reaction expresses the mass of polymer formed. Paclitaxel, 3,4,5-MTS and 2,4,5-MTS added to the reaction mixture at 10 µM concentration increased the polymerized tubulin mass at the steady state level to 142.3, 132.9 and 123.2% of the control value, respectively (Table 2). However, paclitaxel significantly more efficiently shortened the nucleation phase and increased the initial velocity of the polymerization (Fig. 4a, b).

**Table 2:** The effect of 3,4,5-MTS, 2,4,5-MTS and paclitaxel on tubulin polymerization expressed as a percent of control absorbance at steady state level

Concentration (µM)	Steady state level [% of control]		
	3,4,5-MTS	2,4,5-MTS	Paclitaxel
5	113.4 ± 3.6	106.3 ± 4.2	–
10	132.9 ± 17.1	123.2 ± 4.5	142.3 ± 9.2

The results are mean of two independent experiments performed in duplicate ± SEM

### Molecular docking

**Fig. 5:**
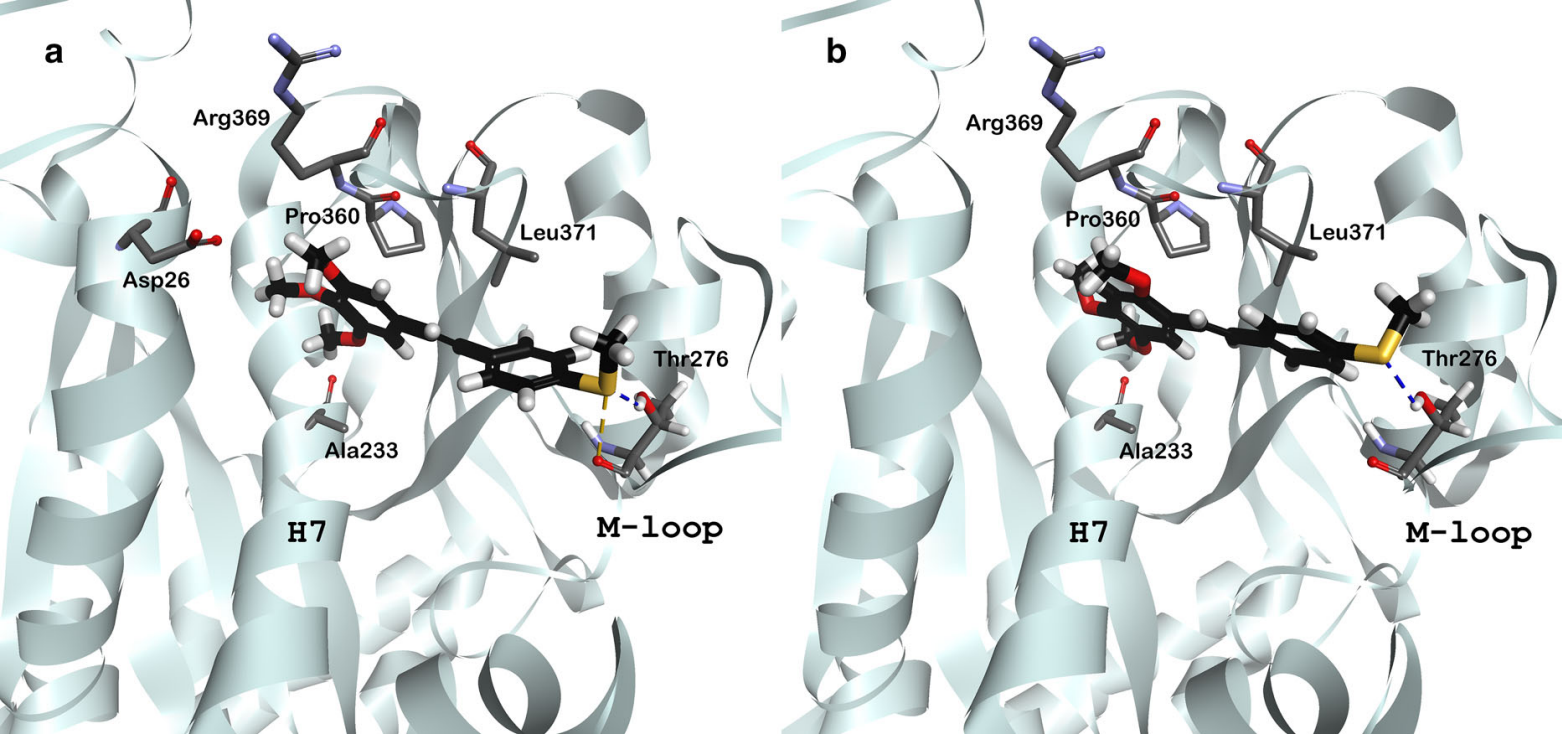
Representative binding modes of 3,4,5-MTS (**a**) and 2,4,5-MTS (**b**). Both *trans*-stilbene derivatives bind between helix H7 and M-loop forming hydrogen bond with Thr276 (blue dashed line). For 3,4,5-MTS sulfur atom enables additional attractive nonbonded S···O interaction with carbonyl oxygen atom of Thr276 (orange dashed line). (Color figure online)

We used a molecular docking approach to elucidate whether the influence of the analyzed molecules on tubulin polymerization is due to a specific interaction with paclitaxel binding site of β-tubulin. 3,4,5-MTS and 2,4,5-MTS were successfully docked to the appropriate binding site of tubulin (PDB: 1JFF) by means of the CDOCKER procedure. The predicted binding modes for both molecules were very similar; they bind to the domain localized between helix H7 and M-loop. As shown in Fig. 5, the analyzed ligands interact with Pro360 and Leu371 (π-alkyl interactions), Ala233 (weak hydrogen bond), Arg369 (amide π-stacked interaction) and form a conventional hydrogen bond with Thr276. However, 3,4,5-MTS has a slightly different orientation, which allows for additional contact with Asp26 and specific S…O interaction with the carbonyl oxygen atom of Thr276. The sulfur atom in methylthio substituent acts as a hydrogen bond acceptor and also enables an attractive nonbonded S···X (X=O) interaction with Thr276 of β-tubulin. CDOCKER interaction energies calculated for 3,4,5-MTS and 2,4,5-MTS were –31.4 and –33.1 kcal/mol, respectively. The representative poses of docked compounds in the paclitaxel binding site are shown in Fig. 5.

## Discussion

The cell-cycle dysregulation and inhibition of apoptosis are critical in the growth and development of neoplasms. Thus, these two events, along with interference with microtubule assembly/disassembly, became attractive targets toward cancer chemoprevention and therapy (Athar et al. 2009; Vang et al. 2011). In this study, we investigated the cytotoxic effect of resveratrol and its methylthio-derivatives with the use of cell lines derived from breast epithelium, non-tumorigenic MCF12A and tumorigenic MCF7 and two cell lines derived from human skin epidermis, spontaneously immortalized HaCaT and epidermoid carcinoma A431 cells. The results of screening studies prompted us to investigate the proapoptotic activity of two methoxy derivatives of 4′-methylthio-*trans*-stilbene: 3,4,5-MTS and 2,4,5-MTS in comparison with the effect of *trans*-resveratrol. By comparing the cytotoxicity of 3,4,5-MTS and 2,4,5-MTS, we found a significantly higher cytotoxic activity of these compounds against MCF7 versus MCF12A, whereas no significant difference in cytotoxicity against A431 and HaCaT was observed. This effect was in agreement with the results of Annexin-V binding assay, which demonstrated a significant increase in the number of dead MCF7 cells in comparison with non-tumorigenic MCF12A cells, while in case of HaCaT and A431 cells a negligible difference occurred. In conclusion, the hypothesis that 3,4,5-MTS and 2,4,5-MTS are selectively proapoptotic against tumorigenic cells was proven in human epithelial-like mammary cells, but failed in relation to human normal and malignant keratinocytes.

Our results indicate a proapoptotic effect of resveratrol in all studied cells with significant dose-dependent increase in the number of apoptotic cells in tumorigenic MCF7 and A431cells. Earlier studies in A431 cells have shown that resveratrol treatment resulted in cell growth inhibition, G0/G1-phase cell cycle arrest and induction of apoptosis (Ahmad et al. 2001; Madan et al. 2008). Thus, our study basically confirmed these observations. Moreover, the proapoptotic effects were observed at doses lower than that usually studied for resveratrol in in vitro experiments (Scott et al. 2012). However, as assessed by flow cytometry, in MCF12A, MCF7 and A431 cells, resveratrol blocked the cell cycle in S-phase. Our results confirm the cell cycle block at S-phase and apoptosis induction in MCF7 cells reported earlier (Pozo-Guisado et al. 2002) and the particular sensitivity of MCF7 line to cytotoxic effect of resveratrol (Li et al. 2006).

In contrast to A431 cell line, normally differentiated non-tumorigenic epithelial HaCaT cells treated with resveratrol were arrested in G0/G1-phase. Therefore, it is possible that in HaCaT cells resveratrol upregulates p53, leading to G0/G1 phase extension. Our results are consistent with the recent studies (Chu et al. 2017) which demonstrated the arrest in G1 stage of cell cycle in HaCaT cells by epigallocatechin gallate.

A431 cells have a nonfunctional (mutated) *TP53* gene. In response to DNA damage, protein p53 triggers a variety of cell-regulatory events to limit the proliferation of damaged cells (Amaral et al. 2010). However, in some cell lines apoptosis induced by resveratrol was evidently p53 independent (Pozo-Guisado et al. 2002; Yuan et al. 2015; Mahyar-Roemer et al. 2001). The studies of Gogada et al. (2011) demonstrated that resveratrol is an inducer of Bax-mediated caspase activation, cytochrome c release and apoptosis in cancer cells, which lack functional p53. Taking into account many biological activities of resveratrol it is presumed that resveratrol activates different pathways and finally the experimental observations may be a consequence of the resveratrol impact on multiple proapoptotic targets. However, the molecular mechanism of resveratrol-induced apoptosis may be different in cancer cells representing different malignancies, additionally displaying different redox status (Benitez et al. 2007; Bresgen et al. 2010; Hecht et al. 2016). Moreover, the ERα status of studied cells may be important in studies of cell specific effect of chemopreventive agents on cell proliferation (Chin et al. 2015).

In contrast to the parent compound, its methylthio derivative 3,4,5-MTS exhibited a more specific effect toward both MCF7 and A431 cancer cell lines, showing the dose-dependent increase of the percentage of apoptotic cells and causing G2/M phase cycle arrest. Although such tendency was observed also in normal immortalized cells, this effect was more significant in cancer cells, particularly at the highest dose. Moreover, similarly to paclitaxel, 3,4,5-MTS derivative promoted microtubule polymerization in vitro. It can be assumed, therefore, that by interacting with tubulin this compound dysregulates mitotic spindle formation and induces mitotic arrest and apoptosis. These results indicate that the pattern of 3,4,5-trimethoxy phenyl may be particularly important for cell cytotoxicity and mechanisms involved in it. Such an assumption is further supported by the fact that DMU-212 (3,4,5,4′-*trans*-tetramethoxystilbene) possessing 4′–methoxy group, extensively studied over the last 10 years, is considered to be the most promising polymethoxy-*trans*-stilbene derivative (Cichocki et al. 2014). In this regard, it was shown that DMU-212 exerts a stronger anti-proliferative and pro-apoptotic effect than resveratrol in ovarian cancer cells in vitro (Piotrowska et al. 2012). Similar effects were also found in other cell systems including human breast cancer cells HCA7 and MCF7 (Ma et al. 2008; Sale et al. 2005; Androutsopoulos et al. 2011). In the latter cells, DMU-212 exhibited submicromolar toxicity after a 96 h exposure, while 3,4,5-MTS inhibited a proliferation of MCF7 cells with IC_50_ of 2.07 µM after a 48 h exposure to the test compound.

As shown in Annexin-V assay, 3,4,5-MTS and 2,4,5-MTS in the range of the concentrations used induced apoptosis in all the studied cell lines. However, these results were not fully supported by TUNEL assay which determines the number of sites of free 3′-OH DNA ends yielded during the apoptotic process. In the studied range of concentrations, a significant dose dependence for 3,4,5-MTS and 2,4,5-MTS was not demonstrated. However, an increase in the number of TUNEL positive cells in relation to several concentrations of the studied compounds was observed in MCF12A, HaCaT and A431 cells. Nonetheless, in compliance with Annexin-V assay, the effect of resveratrol on the number of TUNEL positive cells was statistically significant in all the cell lines studied.

The effect of stilbenes on the process of tubulin polymerization depends on the *cis* or *trans* structure of compounds. Polymethoxy derivatives of *trans*-stilbene are found to enhance tubulin polymerization, contrary to *cis*-stilbenes which exhibit a microtubule-inhibiting effect (Kingston 2009; Tron et al. 2006; Mikstacka et al. 2013). More recently, Scherzberg et al. (2015) observed the inhibitory effect of *cis*-3,4,5-trimethoxystilbene on tubulin polymerization with the immunofluorescence staining method, while *trans*-resveratrol did not affect the process. Their results indicate that methylation of the hydroxyl groups is a critical modification influencing the antitubulin effect. The opposed effect of *cis* and *trans* isomers on microtubule dynamics relies on different sites of their interaction with αβ-tubulin heterodimers; *cis*-stilbenes link preferentially to the colchicine binding site, which is located at the interface between subunits of the tubulin dimer (Ravelli et al. 2004), while *trans*-stilbenes demonstrate affinity to the paclitaxel binding site. This site is located in a deep hydrophobic pocket on the β-tubulin (Nogales et al. 1995). However, the effect of *trans*-stilbenes on tubulin polymerization might be dependent on factors other than a specific binding of compounds to tubulin. That is why we employed molecular docking in order to evaluate the putative interactions between the studied stilbenes with tubulin. In fact, 3,4,5-MTS and 2,4,5-MTS interacted with amino acid residues of the paclitaxel binding site with interaction energies – 33.1 and – 34.6 kcal/mol, respectively, whereas for the paclitaxel molecule the interaction energy was – 71.8 kcal/mol. It must be noted that, owing to its size and complex structure, the molecule of paclitaxel may bind more tightly in comparison with planar molecules of stilbenes.

With the use of molecular docking, we confirmed the results of our experimental studies, demonstrating the stabilizing effect of 3,4,5-MTS and 2,4,5-MTS on tubulin polymerization, although the effect of the studied stilbenes was weaker than that observed for paclitaxel. Molecular docking revealed interactions of the studied derivatives with critical residues of the paclitaxel binding site: Pro360, Leu371, Asp26, Arg369, Ala233 and Thr276. It is noteworthy that for 3,4,5-MTS the interaction of the 4′-methylthio group with Thr276 consisted of a hydrogen bond of sulfur with the hydroxyl group of Thr and an attractive nonbonded S···O interaction with the carbonyl oxygen atom (Fig. 5a).

According to the studies by Ma and coworkers (2008), resveratrol does not influence tubulin polymerization, while the effect of DMU-212 on microtubule polymerization at the dose of 2.5 μM was comparable to that of paclitaxel at the dose of 10 μM. Thus, the contribution of the tubulin-interfering activity of polymethoxy *trans*-stilbene derivatives in their cytotoxic action might be important and characteristic of this group of anticancer agents. However, apoptotic cell death induced by tubulin-interfering agents may occur also via a signaling pathway independent of microtubules and G2/M arrest. Moreover, in our study Annexin-V/PI assay showed in MCF7 cells a significant increase in the percentage of necrotic cells as a result of treatment with 3,4,5-MTS. This effect might result partly from the fact that these cells do not express casapase-3, which is crucial for the induction of apoptosis. The lack of caspase 3 may affect apoptotic response in MCF7. However, these cells are still sensitive to cell death induction (Janicke 2009), and their apoptotic death may proceed via a sequential activation of caspases 9, 7 and 6 (Liang et al. 2001). The contribution of tubulin polymerization impairment in the mechanism of cytotoxic action of 3,4,5-MTS should be investigated further.

## Conclusion

Our results indicate for the first time that the position of the methoxy group in methylthio-stilbenes is critical for the cell cytotoxicity of these compounds and mechanisms involved in it. Moreover, our results support the hypothesis that a strong cytotoxic effect of 3,4,5-MTS, particularly in MCF7 and A431 cells, is due to apoptosis induced by G2/M phase arrest. Both resveratrol analogs 3,4,5-MTS and 2,4,5-MTS differ significantly in their cytotoxic and anti-proliferative activities, even though their effect on tubulin polymerization is comparable. Although microtubule interfering activity is relevant, there must be other essential factors influencing the proapoptotic activity of these compounds.

Our study showed that 3,4,5-MTS may be considered a new lead compound which affects tubulin polymerization causing the cell cycle G2/M –phase arrest followed by apoptosis. Further mechanistic studies on the expression of proteins regulating apoptosis and, in a longer term, pharmacologic studies in animal models could determine the usefulness of 3,4,5-MTS as an anti-cancer agent.
